# Multiple time-scales and the developmental dynamics of social systems

**DOI:** 10.1098/rstb.2011.0214

**Published:** 2012-07-05

**Authors:** Jessica C. Flack

**Affiliations:** 1 Center for Complexity and Collective Computation, Wisconsin Institute for Discovery, Madison, WI 53715, USA; 2 Santa Fe Institute, Santa Fe, NM 87501, USA

**Keywords:** multi-scale organization, social niche construction, networks, signalling, conflict, social complexity

## Abstract

To build a theory of social complexity, we need to understand how aggregate social properties arise from individual interaction rules. Here, I review a body of work on the developmental dynamics of pigtailed macaque social organization and conflict management that provides insight into the mechanistic causes of multi-scale social systems. In this model system coarse-grained, statistical representations of collective dynamics are more predictive of the future state of the system than the constantly in-flux behavioural patterns at the individual level. The data suggest that individuals can perceive and use these representations for strategical decision-making. As an interaction history accumulates the coarse-grained representations consolidate. This constrains individual behaviour and provides the foundations for new levels of organization. The time-scales on which these representations change impact whether the consolidating higher-levels can be modified by individuals and collectively. The time-scales appear to be a function of the ‘coarseness’ of the representations and the character of the collective dynamics over which they are averages. The data suggest that an advantage of multiple timescales is that they allow social systems to balance tradeoffs between predictability and adaptability. I briefly discuss the implications of these findings for cognition, social niche construction and the evolution of new levels of organization in biological systems.

## 1. Introduction

The origins of social complexity have long fascinated anthropologists, sociologists and biologists [1–5]. Attempts to classify social structures by their complexity remain largely qualitative. Two reasons for this are that there is little agreement about what constitutes social complexity and there are few formal complexity measures that work well on real-world data [6,7]. One way to ground the discussion of what social complexity is and how to measure it, is to study how functionally significant aggregate properties arise from microscopic dynamics. Such an approach can reveal the natural scales of the system and the types of structure associated with those scales [8]. This can clarify the kinds of formal complexity measures [9] that might be usefully applied to measure ordered states at different levels of social organization. In addition, before one asks about complexity at the macroscopic scale, one might ask why there are multiple scales at all.

In this paper, I review a body of work on the developmental dynamics of pigtailed macaque social organization and conflict management. This work suggests that ‘higher organizational levels’ in social systems arise when coarse-grained representations of interactions at lower levels become useful to individuals for decision-making. The time-scales on which these representations change are a function of their ‘coarseness’ and the collective dynamics over which they are averages. The data further suggest that multiple time-scales allow social systems to balance tradeoffs between predictability and adaptability.

## 2. Background

Little is known about the functional diversity of social network structures or how functionally significant, aggregate social properties encoded in these networks arise. We also know little about the time-scales on which these social structures and their associated statistical properties change—hence the extent to which social structure can influence behaviour through feedback. These are questions about the construction or development of social systems and, more generally, pattern formation and collective behaviour.

The well-developed body of work on the evolution of institutions [10,11] might, in principle, seem relevant to these developmental questions. In practice, however, studies of institutions rarely address issues of construction of complex aggregate social traits. Instead, ‘institution’ more often than not is a code word for counts or ratios of strategies in a given equilibrium distribution. Although simplifying the problem of institutions in this way makes models tractable and may be justifiable in some cases, it is not fully satisfactory. Many of the institutions observed in human and other social systems have a more complicated statistical character and this needs explaining.

When the models and statistics used to operationalize an institution or aggregate social property are not just counts over strategies but require a more elaborate computation, and when the inputs are not simply individual traits (cooperate, defect, etc.) but network data, then we need to consider explicitly the mapping between behavioural strategies at the individual level and social organization [6]. How do these strategies get collectively combined by multiple individuals to produce aggregate social properties? How much degeneracy characterizes this mapping? Once we can describe how an aggregate social property is produced, we can study how the social process producing it might have evolved.

These kinds of questions should be familiar to readers who know the history of the debate in evolutionary theory surrounding the genotype–phenotype map (for review, see [12]). Two long-standing assumptions in population genetics are that the g-p map, as it is called, is simple, and that the time-scale on which the environment changes is slow enough compared with evolutionary (or behavioural) change that it can be treated as static (the adiabatic assumption).

We now know that the first assumption is wrong for most organisms—the gene activation patterns underlying phenotypic traits are modulated by complex regulatory machinery that itself evolves—the work of Eric Davidson and co-workers on echinoderm development stands as an excellent example [13,14]. And, the second assumption, which if correct would justify studying development and evolution independently, is problematic in any system in which organisms (or components) can modify environmental variables and by modifying them change the selection pressures to which they are subject, as in ecological [15,16] and social niche construction [17]. The consequences of softening these assumptions are now being explored by researchers who work in the evolution of development and related fields like epigenetics. With these advances, we are seeing the beginnings of an evolutionary theory that can account for the origins and diversity of complex forms, as well as for causes of gene change [18].

The role of developmental dynamics has long been debated in the larger evolutionary theory, and so research programmes emphasizing developmental mechanisms have been pursued in parallel to population genetics. Hence the current merger of development and evolution was in a way poised to happen as the data to give momentum to the merger have been (partly) collected. In social evolution, on the other hand, there has only been the game-theoretic-population-genetics trajectory with no sizable quantitative research programme on the developmental dynamics of social organization running in parallel [19] (an exception is the work on social insect societies). Consequently, social evolution lags behind the larger evolutionary theory in its quantitative progress understanding the origins and diversity of complex societies.

With the goal of illustrating the kinds of data and studies that are needed to address developmental questions, I review here what we know about the developmental dynamics of social organization from studying the consolidation of power structure in macaque societies.^1^ My collaborators and I use pigtailed macaque (*Macaca nemestrina*) society as a model system for studying how aggregate social properties arise because functionally significant course-grained information about fighting ability has been shown to be encapsulated in a distribution of power [21–23].

In this system, power—the degree of consensus among group members that an individual can use force successfully during a fight—predicts the cost of social interaction. The data I review below suggest individuals use this information to choose among social interaction strategies. In addition, heavy-tailed distributions make accessible intrinsically costly robustness and conflict management mechanisms like third-party policing, by reducing the cost of these strategies to negligible for individuals in the tail of the distribution [24]. Whereas power reduces uncertainty about the cost an individual is likely to pay during social interactions and for some individuals the cost of social interaction, policing reduces the average cost of social interactions [25]. Together policing and power facilitate the construction of integrated social networks that afford individuals more reliable access to a greater diversity of social resources such as coalition partners and knowledgeable individuals [17,25].

As the studies cited above illustrate, power in pigtailed macaque society is a critical social variable. However, power is not a simple variable. The distribution of power does not map directly onto a distribution of body sizes or even a distribution of fighting abilities. Rather, it is constructed collectively as individuals learn about one another's fighting abilities and signal about this to reduce social uncertainty. In §3, we review data on the process by which power structure arises from aggression dynamics.

## 3. Case study: consolidation of power structure in a primate society

The data used in our case study come largely from studies of conflict dynamics in a group of socially housed pigtailed macaques kept at the Yerkes National Primate Center in Lawrenceville, Georgia. Study system details and data collection protocols, as well as operational definitions, can be found in detail in the methods sections of the work cited and in brief in §6 of this paper.

We have defined an individual's social power as the degree of consensus among group members that it can use force successfully during agonistic interactions [21,26]. Consensus is important when interactions are not strictly pairwise but can involve multiple individuals. In the pigtailed macaque study group, as we will review, information about power is encoded in a subordination status-signalling network. A power distribution consolidates as each individual integrates over its incoming signals to estimate how it is perceived by the group [21,23]. This signalling network arises in turn from an underlying aggression or fight outcome network. Hence multiple, hierarchically organized networks underlie the distribution of power. We sketch below the process generating each network and, ultimately, the power structure, reviewing the existing literature and supplementing these details with new results.

### (a) Fight outcome network

*Individuals interact*. An interaction is any event in which there is an opportunity for immediate contact or in which a signal has been exchanged. Interaction patterns vary across time, such that at any given moment, some fraction of group members is in contact, proximity or signalling from afar. Some of these interactions are fights for dominance and other resources. Whether individuals win or lose fights depends on (reviewed in the studies [22,27]) *temporally stable factors*—including body size, fighting experience and size of alliance networks. *Contextual factors*, including fatigue, variation in priorities, leverage [28], the presence of coalition partners and immediate past successes or failures in fights are also important in so far as they generate stochasticity in fight outcomes, but it is temporally stable factors that predict who will be the winner on average.

In our analyses, the number of nodes in the study group's fight outcome network is given by the number of socially-mature individuals, *n* = 48. The number of potential edges, or agonistic dyads, is (48 × 48)–48 or 2256 (in these analyses we do not consider self-loops). Of those 2256 dyads, 761 dyads were observed to fight (here meaning that at least one individual in the dyad directed aggression at the other) at least once during the study period (data collection details provided in §6). The mean number of agonistic interactions per individual (as either the initiator or initial recipient) was 2.11 (s.d. = 0.713). The mean is low because the fight outcome matrix is relatively sparse.

An individual can win, lose or draw in these interactions. For the reasons described above, the outcome of interactions can vary in time. The directional consistency index for the outcome of fights in the study group is 90.16 per cent. The directional consistency index [29] is calculated by summing over all dyads, the absolute value of wins minus losses for each dyad, divided by the total number of encounters for all individuals. It is a measure of the consistency of outcomes in aggregate (over all observed fights between a pair considered over all dyads) and hence provides a crude measure of stochasticity.

By constructing a fight outcome time series for each pair of individuals, we can determine for that pair the number of times that fight outcome changes over sequential fight bouts from win to lose to draw, etc. This allows us to determine the extent to which fight outcomes vary temporally, from fight to fight.

Of the 761 dyads that were observed to fight, fight outcome (edge directionality in the fight outcome network) changed 292 times. For these dyads, the mean number of outcome changes per dyad was 0.384 (s.d. = 0.9285) and the mean number of changes per dyad per hour was 0.003. The maximum number of outcome changes observed per dyad was 11 and the minimum was 1. Although the directional consistency index suggests that fight outcomes are fairly consistent in aggregate, the time-series data reveal the presence of fight-to-fight fluctuations. Hence reliance on any single outcome as a predictor of future outcomes for decision-making can be misleading.

### (b) Subordination signalling network

Once a pattern of losing exceeds a certain threshold, the individual perceiving itself as likely to lose signals this recognition using a bared teeth (BT) display [27,30–32]. In pigtailed macaques, BTs are emitted in two contexts: fights and peaceful interactions in which one individual passes by or approaches the other, showing no threatening behaviour [32]. Both displays in our pigtail macaque group are highly unidirectional, meaning the same individual emits the signal nearly 100 per cent of the time until the perceived asymmetry in fighting ability is deemed by the signaller to no longer hold (peaceful variant dci: 99.7%; agonistic variant dci: 96.6% [32].

Individuals exchanging peacefully-emitted silent-BT displays have stronger affiliative relationships with one another (more grooming, more reconciliation and less aggression) than those who only exchange the BT display during fights. Multiple analyses described in Flack & de Waal [32] indicate that this relationship is causal: giving silent-BT displays in peaceful contexts improves relationship quality.

**Figure 1.**
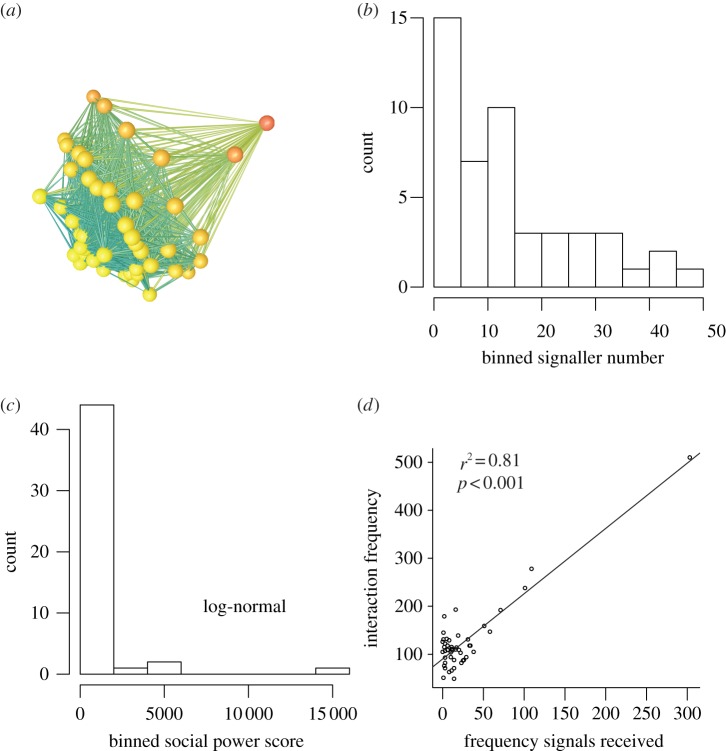
(*a*) Subordination signalling network. Nodes are individuals and are coloured by frequency of signals received (orange node receives largest number of signals). (*b*) Frequency distribution of number of signallers to a given receiver (unweighted in-degree distribution of signaller number). (*c*) Frequency distribution of social power, where power is computed for individual *i* by multiplying *i*'s total number of signals received by its number of signallers (§6). (*d*) Relationship between signals received and interaction frequency (operationalized as signals received plus signals emitted plus fights participated in).

We also find that individuals who receive more peacefully emitted signals have more interactions (figure 1*d*), where an individual's interaction frequency is operationalized to include its subordination signalling events, regardless of whether it is the sender or receiver, and all of its agonistic interactions, regardless of whether it is the initiator or initial recipient. Our previous work [32] suggests that receiving signals increases interaction rate, rather than the other way around.

Whereas the agonistic BT variant signals submission—defined as impending withdrawal in the present interaction, the data in the studies reviewed above suggest the peacefully emitted silent-BT displays are subordination signals that communicate agreement to a primitive social contract in which the signaller agrees for some time period to the subordinate role, and thus to yield when a conflict arises in the future.

The contract is cost-free (originally defined by Smith & Harper [33], see also Bergstrom & Lachmann [34]), as both sender and receiver prioritize the exchange of the signal above not signalling (signalling is the optimal strategy for the individual perceiving itself as the loser) [22,32]. The contract is upheld as long as two conditions are satisfied. The signaller yields when the receiver expresses an interest in a resource, and the underlying asymmetry must continue to be perceived by the subordinate as large. We have proposed that an advantage of the contract is that it establishes a new conditional symmetry, in which the sender and receiver are free to interact (e.g. groom, etc.) with a reduced concern that a fight will erupt—a form of conditional equality [32].

A subordination signalling network, also called a subordination contract network, can be constructed from the pairwise signalling data.^2^ The number of nodes in the subordination signalling network is given by the number of socially-mature individuals. In the case of our study group, again *n* = 48. The number of potential edges, or agonistic dyads in this group, is again (48 × 48)–48 or 2256. Of those 2256 dyads, 704 exchanged at least one peacefully-emitted subordination signal, giving a network with 704 edges. Figure 1*a* shows the signalling network and figure 1*b* the unweighted in-degree distribution (e.g. the number of signallers who signal to node *i*) in the pigtailed macaque study group for a stable 4 month period (empirical methods).

The data suggest it takes several reversals of edges in the fight network before a signal is withheld and many more before signal sender and receiver reverse their contract [31,32,35]. This means that statistical features of the signalling network—for example, the rate of edge flipping and the weighted degree distribution, should be relatively impervious to fluctuations in fight outcomes.

Our data support this conclusion. By constructing a signal exchange time series for each signalling pair, we can determine for each pair the number of times that the direction of signal exchange changes (e.g. individual *i* signalled to *j* at *t_1_* and *j* signalled to *i* at *t*_2_). Of the 704 dyads that were observed to exchange signals, signal direction reversed only twice—once in each of two dyads—over the course of the 4 month study period. The mean number of changes to signal direction per dyad was 0.003 (s.d. = 0.053) and the mean number of changes in signal direction per dyad per observation hour was 0.00002. The maximum number of switches observed per dyad was 1.

Based on these averages, edges in the signalling network change approximately 165 times (roughly two orders of magnitude) more slowly than edges in the aggression network. These data in combination with the directional consistency index for signal direction indicate that subordination signalling is highly deterministic at both the event and aggregate levels, whereas fight outcomes are fairly to highly deterministic in aggregate but fluctuate across fights. The subordination contract encodes coarse-grained, reliable information about fighting ability and consequently a single signalling event is a better predictor of fight outcomes than any single fight outcome is. My collaborators and I have called these kinds of predictive, coarse-grained variables *slow variables* [6].

In summary, an asymmetry in perceived fighting ability builds up at the pairwise level through memory of past outcomes of competitive interactions. Once it becomes clear to one individual in a given pair that it is more likely to lose a fight (e.g. a threshold is passed) with its adversary, the individual perceiving itself as the likely loser emits a subordination signal. This is the optimal strategy if it cannot win—hence the signal is said to be cost-free. Individuals appear to reference the signal exchange, rather that fight outcomes, for strategical decision-making because the signal changes direction more slowly than fight outcomes and is not influenced by transient contextual factors, and so is a more reliable indicator of relationship state. The reduction in the frequency of aggression—and hence the opportunity for reversing the dominance relationship—that follows signal exchange further consolidates the dominance–subordination relationship. However, some level of fighting continues, providing a mechanism for relationship reversal.

### (c) Power structure

Encoded in the subordination signalling network is information about power; that is, how much consensus there is among group members that an individual is capable of successfully using force. Elsewhere [21,23], we have explored the utility of various information theoretic and diffusion-based algorithms for computing consensus on networks about node *i*'s capacity to use force*.* The algorithms take a subordination signalling matrix as input and output a score for each individual. The index of scores gives the distribution of power. Our analyses indicate that individuals take into account how many signals they receive in total from their population of signallers weighted by a measure of the diversity of that signalling population (which can be a simple count of the number of signallers or something more elaborate like the Shannon entropy of the vector of signals received by individual *i*) to estimate how much consensus there is in the group about their capacity to use force, and hence how much power others collectively perceive them to have.^3^

The data suggest that in addition to having the capacity to estimate their absolute power, the individuals in the study system are capable of roughly estimating their relative power, and hence know something about the distribution of power in the group [21,24]. We are presently exploring the heuristics the animals could be using to make such estimates. This is a critical issue, as such aggregate social variables produced from a collective process (many individuals contribute to signal network structure) can only have causal or feedback consequences for individuals in the system if the individuals can make reasonable estimates of those variables from the behavioural sample to which they have access and given their computational capacity.

Figure 1*c* shows the distribution of power in the pigtailed macaque study group. The distribution is not significantly different than log-normal according to the Lilliefors test.

An individual's estimate of its power can predict, when the estimate is a good one, the cost it will pay on average during social interactions, thereby changing the probability of social strategy use, and the accessibility of strategies, like policing, for managing conflict [24].

In general an individual's power score will change more slowly than any one of its contracts because either many of its contracts need to change or many contracts of other individuals need to change, before its power score will change. Although this suggests that power, like the subordination contract, is a slow variable, serving as a reliable reference for decision-making we do not have enough data in the present case study to estimate the precise time-scales on which properties of the power distribution change.

Yet, the time-scale on which power changes is likely to be critically important. Although the power structure must change slowly to be useful for prediction, it cannot change too slowly because it needs to approximately represent the underlying distribution of fighting abilities, and this changes over time [22]. Hence important questions include (a) what is the optimal degree of correlation between the power distribution and the underlying distribution of fighting abilities, (b) how stationary is this correlation, (c) how does the algorithm used to compute power scores from the signalling network influence the time-scale on which features of the power distribution change, and (d) how well can individuals, given their computational capacity, estimate their relative scores given the complexity of topology of the signalling network and the computational steps required to assess consensus?

### (d) Emergence of novel social functions

A positively skewed distribution of power with a long tail (e.g. power-law tail) describes a society in which a non-vanishing minority of individuals are collectively perceived as disproportionately powerful. The power structure in our study group is best described by this kind of heavy-tailed distribution [21]. The data suggest that these power structures can support the implementation of novel, beneficial conflict regulatory mechanisms, such as policing [24]. For any individual to adopt this policing role, it must be able to estimate the cost it will pay for intervening, and that cost must be low. In our study group, this cost is negligible for the individuals in the tail of the power distribution [24].

A behavioural knockout experiment in which the policing mechanism was temporarily disabled by preventing the policers from intervening in fights, showed that policing increases societal robustness to conflict perturbations [17,25]. Knockout resulted in a destabilization of the groups' social networks: the cost of social interaction increased, investment in social capital acquisition, like alliance partners, decreased, and the cliquishness and assortative structure of the group's social networks increased.

Hence in this system a heavy-tailed distribution of power makes intrinsically costly conflict management mechanisms like policing more accessible strategies by reducing their cost for individuals towards the tail of the distribution. Policing in turn reduces the average cost of social interactions for all individuals and so allows for more efficient social, and presumably ecological, resource extraction.

## 4. Main findings of case study

**Figure 2.**
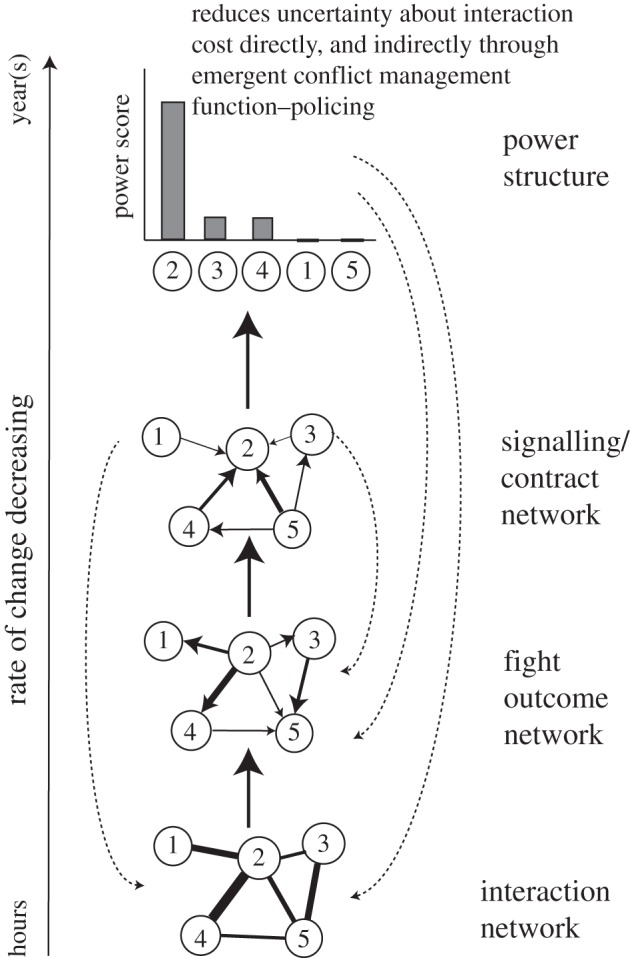
Schematic illustrating the dynamics and proliferation of temporal scales underlying the consolidation of power structure and the emergence of a new conflict management function through the build-up and amplification of asymmetries resulting from competitive interactions among individuals. The dashed arrows represent feedback from consolidating higher levels of organization to lower levels. Solid lines indicate a feed-forward process whereby summary statistics or coarse-grained variables get consolidated as small fluctuations in competitive ability at the individual level are amplified through memory, generating long-lived asymmetries in competitive ability, or as individuals come to learn underlying differences in competitive ability. As a consequence of integrating over abundant microscopic processes, these consolidating summary statistics, which we call slow variables, provide better predictors of the local future configuration of a system than the states of the fluctuating microscopic components. See §3 for further details.

Underlying the consolidation of the power distribution and emergence of policing are several hierarchically organized networks (shown in figure 2 schematically)—an interaction network (which I have not discussed here), a fight outcome network, and a status signalling, or social contract, network.

Our data suggest that the advantage of multiple, hierarchically organized networks are that their variable rates of change can be used to maximize objectives that would be at odds if there was only a single time-scale. Individuals, by temporarily agreeing to a contract, reference the slowly changing contract for strategic decisions concerning the receiver (or dominant partner) rather than the rapidly changing and potentially misleading fluctuations in fight outcomes with that individual. Individuals use information about their relative power encoded in the signalling network to make decisions about how to behave during polyadic conflicts, as described at the beginning of this case study. Allowing a low level of fighting to continue even after contracts are formed and a stable power structure has been established is advantageous because it prevents lock-in by allowing learning to continue. This provides a mechanism by which the social contract can be reversed (and relative power can change) as new asymmetries become established and old ones get modified.

Our analyses suggest that the power structure arises through the collective (across multiple individuals) accumulation of memory of asymmetric, and typically competitive, outcomes. This accumulation of memory is stored in the slowly changing signalling network. As the power structure becomes established, asymmetries at the individual level are amplified through feedback effects. Hence as a history of competitive outcomes builds up, the coarse-grained representations of these microscopic dynamics consolidate around stable values and so become slowly changing predictors of the social system's future state. This reduces social uncertainty by providing a stable social environment against which individuals can tune their behavioural interaction strategies.

## 5. Discussion and broader implications

Statistical properties of the power distribution (e.g. mean power score, variance, rank order) encode information about likely fight outcomes, particularly in multi-party conflicts, and are examples of what we have called *slow variables* [6]. Slow variables, whether social or biological, arise from mechanisms that naturally integrate over fast, microscopic dynamics. Proteins, for example, have a long half-life relative to RNA transcripts, and can be thought of as the summed output of translation. Cells have a long half-life relative to proteins, and are a function of the summed output of arrays of spatially structured proteins. Social power scores in our primate study group have a long half-life compared with individual interactions. Power, proteins and cells—as different as they are—all represent some average measure of the noisier activity of their constituents. As summary statistics for lower level dynamics, these variables can serve as reliable reference states that, when perceivable by system components, can be used by components to make strategical decisions and tune their behaviour.

### (a) Cognitive and computational issues

A critical point I have tried to emphasize throughout the case study is that only *when detectable by the system or its components* can slow variables reduce environmental uncertainty, and, by increasing predictability, promote accelerated rates of microscopic adaptation [6]. Identifying reliable, predictive coarse-grained statistics is a non-trivial problem in most living systems for both observers and the system itself. The system or its components might not have the necessary search or computational capacity. In the case of power, if individuals do not interact frequently enough to build up a history of fights, large asymmetries might not get established or be perceived, and the social contract might not arise as a solution to conflict.

Additionally, the concept of statistical sufficiency [36] tells us that a sizable dataset is required to validate that a posited slow variable is actually a good predictor of the future state of the system. These data are almost never available to observers and are almost certainly not available to components of the system. Hence, if we are to find *natural coarse-grainings* [37,38] of system dynamics, we must work towards formal descriptions that reflect both mathematical rigour and biological parsimony. Formalisms describing how functionally useful aggregate properties are encoded in interaction network data ideally will be cognitively grounded, in so far as the formalisms will take into account factors such as the largest dataset that can be recorded and processed by any component of the system given its cognitive capacity [6].

### (b) Social niche construction as collective slow-variable construction

Social niche construction has been defined as the process whereby individuals in social groups, by edge-building in their social networks, collectively co-construct aggregate level social properties with functional implications for individuals (defined in supplement to Flack *et al*. [17]). Such properties can include the degree of assortativity or clustering characterizing the social networks. These properties have functional implications for individuals if—for example—they constrain strategy choice by limiting who interacts with whom, or if they influence the consequences of strategy choice by affecting how knowledge, emotion, or behaviour flows over networks.

In our macaque study group, power is a collectively constructed social variable with causal implications for the constructors. It has implications for interaction frequency and conflict management, which in turn have been shown to affect how individuals construct edges in their affiliation networks. As I have discussed, these networks provide access to critical social resources, such as alliance and coalition partners, and individuals with information about ecological resources such as the location of food and sleeping sites. In addition, data from baboons suggest that the quality of the social networks constructed by mothers can impact offspring survival [39]. This means that in addition to the direct effects on the constructor, social niche construction can also affect the constructor's offspring.

The dominant emphasis in the niche construction literature has been on the fitness consequences of niche construction for the constructor and its offspring [15] with little attention to the feed-forward construction dynamic. However, the data I have reviewed in this paper indicate that the mapping between constructor behaviour and the constructed variables can be non-trivial and can play a substantial role in determining the character and strength of the feedback effects. This implies that we will be in a better position to study the evolution of the social niche construction once we take seriously the mapping from individual behaviour to social structure, and can describe algorithmically how aggregate social properties are produced.

### (c) The mechanistic causes of new levels of organization

An unanswered question in evolutionary theory is why life organizes itself hierarchically [40–44]. From cells, to organisms, to societies, evolution generates structures nested in space and time. My collaborators and I have proposed that the construction of slow variables—whether subordination contracts, ocean reefs, proteins or cells—reduces environmental uncertainty and represents the first step in the evolution of a multi-scale system [6]. The underlying idea is that multiple timescales can solve the dual problem of informational noise and informational lock-in. As an interaction history accumulates at the lower level, competitive dynamics amplify asymmetries among components and slowly changing, coarse-grained representations of these dynamics become more predictive of the future state of the system than the interactions themselves. A new level of organization arises as components begin using these slowly changing coarse-grained statistics to make strategical decisions. The concordant increase in predictability resulting from reliance on the coarse-grained representations can promote accelerated rates of microscopic adaptation in two ways: by allowing components to fine-tune their behaviour, and by freeing components to search at low cost (because the alternatives are selectively neutral) a larger space of strategies for extracting resources. The in-flux events, on the other hand, allow the system and its components to closely track environmental changes, allowing adaptation and preventing lock-in.

Under this view, integrated, coordinated aggregates are a solution to uncertainty. If correct, this perspective would suggest that emergence of new levels of biological and social organization, and perhaps the major transitions [42], cannot be accounted for without explicit consideration of developmental dynamics underlying multi-scale systems.

## 6. Methods

### (a) Study system

Macaque societies are characterized by social learning at the individual level, social structures that arise from nonlinear processes and feedback to influence individual behaviour, frequent non-kin interactions and multiplayer conflict interactions, the cost and benefits of which can be quantified at the individual level [17,24,25,31,32,45–47]. These properties make the macaque genus and its representative species excellent choices for drawing inferences about critical processes in social evolution as well as for developing new modelling approaches that are intended to apply more broadly.

The dataset, collected by J.C. Flack, is from a large, captive, breeding group of pigtailed macaques that was housed at the Yerkes National Primate Research Center in Lawrenceville, Georgia. The study group had a demographic structure approximating wild populations. Subadult males were regularly removed to mimic emigration occurring in wild populations. The group contained 84 individuals, including four adult males, 25 adult females and 19 subadults (totalling 48 socially mature individuals used in the analyses). All individuals, except eight (four males and four females), were either natal to the group or had been in the group since formation. The group was housed in an indoor–outdoor facility, the outdoor compound of which was 125×65 ft.

Pigtailed macaques are indigenous to Southeast Asia and live in multi-male, multi-female societies characterized by female matrilines and male group transfer upon the onset of puberty [48]. Pigtailed macaques breed all year. Females develop swellings when in oestrus.

### (b) Data collection protocol

During observations, all individuals were confined to the outdoor portion of the compound and were visible to the observer. The 156 h of observations occurred for up to 8 h daily between 1100 and 2000 h over a 20 week period from June until October 1998 and were evenly distributed over the day. Provisioning occurred before observations, and once during observations. The data were collected over a 4 month period during which the group was stable (defined as no reversals in status-signalling interactions resulting in a change to an individual's power score).

Conflict and power (subordination signal) data were collected using an all-occurrence sampling procedure in which the compound was repeatedly scanned from left to right for onset of conflict or the occurrence of silent-BT displays (used to measure power, see §6*c*). The entire conflict event was then followed, including start time, end time and the identity of individuals involved as aggressors, recipients or interveners (see §6*c* for operational definitions). Although conflicts in this study group can involve many individuals, participation is typically serial, making it possible to follow the sequence of interactions. A nearly complete time series of conflict events is available for each observation period.

### (c) Operational definitions

Conflict/fight: includes any interaction in which one individual threatens or aggresses a second individual. A conflict was considered terminated if no aggression or withdrawal responses (fleeing, crouching, screaming, running away and submission signals) occurred for 2 min from the last such event. A conflict can involve multiple pairs if pairwise conflicts result in aggressive interventions by third parties or redirections by at least one conflict participant. In addition to aggressors, a conflict can include individuals who show no aggression (e.g*.* recipients or third-parties who either only approach the conflict or show affiliative/submissive behaviour upon approaching, see [32]). Because conflicts involve multiple players, two or more individuals can participate in the same conflict but not interact directly.

Agonistic dyad: includes interaction between pairs within a conflict. At least one individual in the pair must direct aggression towards the other. Individual *i* is said to be the winner if its opponent, individual *j* exhibits withdrawal-related behaviour (crouching, shrinking, backing away, screaming, running away and emitting a submission signal) in response to the behaviour of *i* and this withdrawal-related behaviour is not superseded by aggressive behaviour towards *i* at any point during the conflict. Individual *i* is said to be the loser if it is the one to show withdrawal-related behaviour. The interaction is said to result in a draw if both *i* and *j* direct aggression towards each other and this aggression is not superseded by withdrawal-related behaviour, or if one individual directs aggression towards the other and the other shows no response.

Subordination signal: the subordination signal in the pigtailed macaque communication repertoire is the peacefully emitted variant of the silent-BT display. BT displays are marked by a retraction of the lips and mouth corners such that the teeth are partially bared. In pigtailed macaques, the SBT occurs in two contexts: peaceful and agonistic SBT, see Flack & de Waal [32]. Signals in both contexts are highly unidirectional. The agonistic SBT encodes submission. The peaceful variant signals agreement to primitive social contract in which the signaller has the subordinate role [32].

Power: the degree of consensus among group members that an individual can successfully use force in social contexts. For details on the algorithms used to compute power, see [21,22]. This is a simplification of an information theoretic formalism based on the Shannon entropy we developed in Flack & Krakauer [21]. We use it here because it gives a good approximation of the power distribution and is a calculation that is easy to compute when analysing models [23].

## Endnotes

1 The quantitative literature on urban scaling covers another set of issues and type of data critical to understanding social system developmental dynamics. Space constraints prohibit coverage here, but see Bettencourt *et al.* [20].

2 Note that although the signal exchange is pairwise, alliances (which add structure) and coalitions (which add stochasticity) can affect the outcomes of fights. Hence hidden in the pairwise exchange is polyadic information.

3 I note that in animal behaviour the standard approach to ordering individuals based on dominance-related behaviour is to construct a dominance hierarchy. There are many well-known algorithms for generating dominance hierarchies. Many of these algorithms order pairs of individuals so that the order minimizes the number of intransitivities in a matrix containing information about dominance interactions (e.g. wins, subordination signals). The resulting rank order is strictly the outcome of pairwise assessments, rather then the outcome of a whole group or population-level assessment, and hence does not necessarily order individuals according to how much consensus there is that they can use force successfully. Even if such approaches could be said to measure consensus, their output is (typically) an ordinal, not scalar, rank order. This means their utility is limited for studying how variance in the distribution of power influences interaction cost, conflict management, or other social properties that are expected to vary as a function of moments of the distribution of power. I note, however, that in social systems with a normal distribution of fighting abilities and perfect learning, there should be no intransitivities in dominance relationships and the distribution of power should best be described by a uniform distribution that can be effectively captured by an ordinal rank order.
